# Local delivery of FK506 to a nerve allograft is comparable to systemic delivery at suppressing allogeneic graft rejection

**DOI:** 10.1371/journal.pone.0281911

**Published:** 2023-03-07

**Authors:** Brett Davis, Susan Wojtalewicz, Sierra Erickson, Jacob Veith, Andrew Simpson, Himanshu Sant, Jill Shea, Bruce Gale, Jay Agarwal

**Affiliations:** 1 Department of Surgery, University of Utah, Salt Lake City, Utah, United States of America; 2 Department of Mechanical Engineering, University of Utah, Salt Lake City, Utah, United States of America; University of Toronto Temerty Faculty of Medicine, CANADA‬‬

## Abstract

The objective of this study was to determine if locally delivered FK506 could prevent allogeneic nerve graft rejection long enough to allow axon regeneration to pass through the nerve graft. An 8mm mouse sciatic nerve gap injury repaired with a nerve allograft was used to assess the effectiveness of local FK506 immunosuppressive therapy. FK506-loaded poly(lactide-co-caprolactone) nerve conduits were used to provide sustained local FK506 delivery to nerve allografts. Continuous and temporary systemic FK506 therapy to nerve allografts, and autograft repair were used as control groups. Serial assessment of inflammatory cell and CD4+ cell infiltration into the nerve graft tissue was performed to characterize the immune response over time. Nerve regeneration and functional recovery was serially assessed by nerve histomorphometry, gastrocnemius muscle mass recovery, and the ladder rung skilled locomotion assay. At the end of the study, week 16, all the groups had similar levels of inflammatory cell infiltration. The local FK506 and continuous systemic FK506 groups had similar levels of CD4+ cell infiltration, however, it was significantly greater than the autograft control. In terms of nerve histmorphometry, the local FK506 and continunous systemic FK506 groups had similar amounts of myelinated axons, although they were significantly lower than the autograft and temporary systemic FK506 group. The autograft had significantly greater muscle mass recovery than all the other groups. In the ladder rung assay, the autograft, local FK506, and continuous systemic FK506 had similar levels of skilled locomotion performance, whereas the temporary systemic FK506 group had significanty better performance than all the other groups. The results of this study suggest that local delivery of FK506 can provide comparable immunosuppression and nerve regeneration outcomes as systemically delivered FK506.

## Introduction

Peripheral nerve injuries can lead to permanent loss of motor and sensory function and debilitating chronic pain with less than 50% of patients achieving adequate functional recovery after peripheral nerve injury and repair [1–3]. Peripheral nerve injuries that have gaps larger than 3cm often lead to the worse patient outcomes and clinicians have limited efficacious treatment options for the repair of these injuries [4–8]. Nerve autografts are still the gold standard for treatment of nerve gap injuries over 3cm; however, autograft disadvantages limit their usefulness in the clinic. Autografts are in limited supply and can only be harvested from a few sites. Autografts require an additional surgical site and their harvest can result in donor site functional morbidity, painful neuroma formation at the donor site, and result in fascicular mismatching when grafted [9–12]. The limitations of the autograft have spurred decades of peripheral nerve regeneration research to develop an alternative treatment strategy to the autograft. As a result, clinically available alternatives include simple bioresorbable nerve guide conduits and decellularized allografts. Bioresorbable nerve conduits provide gross guidance of regenerating axons but are only recommended for use in gaps less than 2cm [5, 13, 14]. Nerve conduits do not provide the native nerve microstructure, biochemical cues, and Schwann cell support necessary for long-gap nerve regeneration [5, 14]. Decellularized allografts provide the native topological and chemical cues to guide axon regeneration and have been proven to be more efficacious than nerve conduits, but they are still inferior to the autograft in long-gap scenarios greater than 3cm [15, 16]. The lack of translation of technologies that could match or surpass the efficacy of the autograft has left a significant clinical need unmet.

Clinical reports of nerve allograft treatment have been scarce, even after the advent of microsurgical techniques and immunosuppressant drugs in the 1980s [17]. Allogeneic nerve grafts are a potential solution to the limitations of the autograft, but long-term systemic immunosuppression is required for the treatment strategy to be viable [10, 18]. Long-term systemic delivery of FK506 is associated with increased risk of infection, kidney toxicity, and liver toxicity [19, 20]. The harsh side-effects associated with systemic immunosuppression outweigh the benefits in most nerve repair scenarios, which prevent nerve allotransplantation from widespread clinical adoption.

Several studies have shown in both animal models and limited clinical case reports that temporary systemic immunosuppression (e.g. FK506) can prevent allogeneic nerve graft rejection and that nerve regeneration can reach similar levels to the autograft [21–27]. These studies conclude that once regenerating axons have passed through the nerve graft and reached end-targets, immunosuppressive therapy can be withdrawn and functional recovery will be maintained after an acute rejection phase [21, 22, 24, 25]. Little is known about locally delivered FK506 as an effective immunosuppressant to prevent allogeneic graft rejection. Recent reports of local delivery of FK506 to vascularized composite allograft transplants suggest that local delivery of FK506 can produce local immunosuppressant effects [28–35]. However, less is known about local delivery of FK506 to nerve allografts. Temporary local delivery of FK506 may provide temporary immunosuppression to the nerve allograft, allowing regenerating axons to pass through, without the deleterious systemic side-effects. If proven effective, local delivery of FK506 can be a potential solution to the nerve allograft problem.

We hypothesized that temporary local delivery of FK506 to an allogeneic nerve graft could prevent allogeneic graft rejection and allow nerve regeneration to proceed through the allogeneic nerve graft. In previous work, we developed an FK506-delivering nerve conduit that provides sustained local delivery of FK506 for at least 2 months [36, 37]. In the present study, we investigated if the FK506-delivering nerve conduit could provide local immunosuppression and nerve regeneration in a mouse allogeneic nerve graft model. We serially assessed the immune response to the nerve grafts by measuring the CD4+ lymphocyte and inflammatory cell infiltrate into the nerve allografts. We assessed nerve regeneration by quantitating myelinated axons distal to the graft, measuring gastrocnemius muscle mass recovery, and evaluated functional recovery via the skilled locomotion ladder-rung assay.

## Methods

### FK506-loaded nerve conduit fabrication

A depot FK506 delivery system in the shape of a nerve conduit was created by embedding FK506 into poly(L-lactide-ε-caprolactone) (PLC) following a previously described protocol [36]. 10% w/v PLC solutions were made by dissolving PLC (Corbion, Amsterdam, Netherlands) in dichloromethane (Acros Organics, Geel, Belgium). FK506 (PROGRAF, Astellas Pharma., Tokyo, Japan) was dissolved in 100% ethanol and added to the PLC solution to form a solution with final FK506 concentration of 0.03% w/w (FK506/PLC). Drug-loading concentration was chosen based on clinical therapeutic dosage windows and previous drug release studies to maintain a local FK506 concentration between 5-20ng [36, 38, 39]. Polymer films were formed by solvent-casting 13 mL of the PLC/FK506 solutions into glass petri dishes. Films were cured for 48 h in a fume hood followed by an additional 48 h in a vacuum. Films were cut using scissors to 10 × 3.5 mm dimensions. Using a microtipped soldering iron, the long edges of the films were fused together around a glass tube to create a 10mm long conduit.

### Experimental design and in vivo model

The in vivo study protocols were executed as approved by the Institutional Animal Care and Use Committee of the University of Utah. 94 adult male Tg(Thy1-YFP)16Jrs (Jackson Laboratory, Bar Harbor, ME, USA) mice were used in the experimental groups and 33 adult male BALB/c mice were used as allogeneic sciatic nerve donors (Charles River Laboratories, Wilmington, MA, USA). These two strains of mice were chosen because they have differences in major histocompatibility complex class I and class II molecules. The experimental mice were divided into experimental groups: Autograft (n = 29), Allograft + Local FK506 (n = 29), Allograft + Temporary Systemic FK506 (n = 7), and Allograft + Continuous Systemic FK506 (n = 29). In all animals, an 8mm sciatic nerve gap was created and repaired by either an 8mm autologous or allogeneic sciatic nerve graft. The Autograft group, received a nerve autografts without any additional drug or device treatment. The Allograft + Local FK506, received a nerve allograft that was placed through the inner lumen of the FK506-loaded nerve conduit. The Allograft + Continuous FK506 and Allograft + Temporary Systemic FK506 groups received a nerve allograft and daily subcutaneous injections of FK506; however, daily injections were discontinued after 6 weeks for the Temporary Systemic FK506 group. FK506 solutions for injection were created by diluting clinical grade intravenous FK506 solution (5mg/ml PROGRAF, Astellas Pharma, Tokyo, Japan) into 0.9% sodium chloride to a final concentration of 1.667 mg/ml. For the serial assessment of immune response and nerve regeneration, n = 5 mice from each group were sacrificed at weeks 1, 2, and 4, and n = 7 mice from each group were sacrificed at weeks 6 and 16. The Temporary Systemic FK506 group only has n = 7 animals, which were sacrificed at week 16. The mice sacrificed at weeks 1, 2, and 4 underwent nerve tissue harvesting for histologic assessment of the immune response to the nerve graft, and mice sacrificed at weeks 6 and 16 also underwent functional ladder rung testing and gastrocnemius muscle mass recovery evaluation. Nerve histomorphometry was performed only at weeks 4, 6, and 16 since nerve regeneration prior to 4 weeks is minimal.

### Surgical procedure

The rodent study protocols were executed as approved by the Institutional Animal Care and Use Committee of the University of Utah. The mice were housed according to the IACUC and University guidelines at the University of Utah. Mice were anesthetized with isoflurane. A longitudinal incision was made in the posterior distal thigh of the hind limb, separating the natural muscle planes. In the autograft group, the sciatic nerve was isolated and transected immediately distal to the sciatic notch and proximal to the peroneal and tibial bifurcation, for a total length of 8mm. The proximal and distal ends of the isograft was flipped and it was sutured back into the transected ends of the intact sciatic nerve were then repaired using 9–0 nylon epineural sutures. For the allograft group, the sciatic nerve was transected immediately distal to the sciatic notch and an 8mm portion of the nerve was removed. An 8 mm sciatic nerve allograft from a donor BALB/c obtained in a similar fashion was sutured to the transected ends of the host animal using 9–0 nylon epineural sutures. In the Allograft + Local FK506 group, the allograft was placed through the inner lumen of the FK506-loaded nerve conduit. Animals were sacrificed according to the timeline mentioned above for tissue harvest. Animals were provided daily carprofen postoperatively according to IACUC guidelines to alleviate pain. Animals were sacrificed via cervical disolocation under isoflurane anesthesia.

### Skilled locomotion assessment of functional recovery: Ladder rung assay

The ladder rung assay evaluates an animal’s ability to perform skilled locomotion, which is correlated with sensorimotor functional recovery [40]. The animals in the week 6 and week 16 timepoints were trained to cross a horizontal ladder for a period of 2 weeks prior to surgical procedure. At 4, 10, and 16 weeks postoperatively the animal’s ladder rung performance crossing the ladder 3 consecutive times at each timepoint was video-recorded and later analyzed by a trained observer. The number of missteps by the right hindlimb (e.g. the mouse would completely miss a rung or hit the rung but the paw would completely slip off) and total number of steps by the right hindlimb are counted and the slip ratio % is calculated by (# missteps /# total steps) x 100.

### Histological assessment of inflammatory cell infiltration

**Fig 1:**
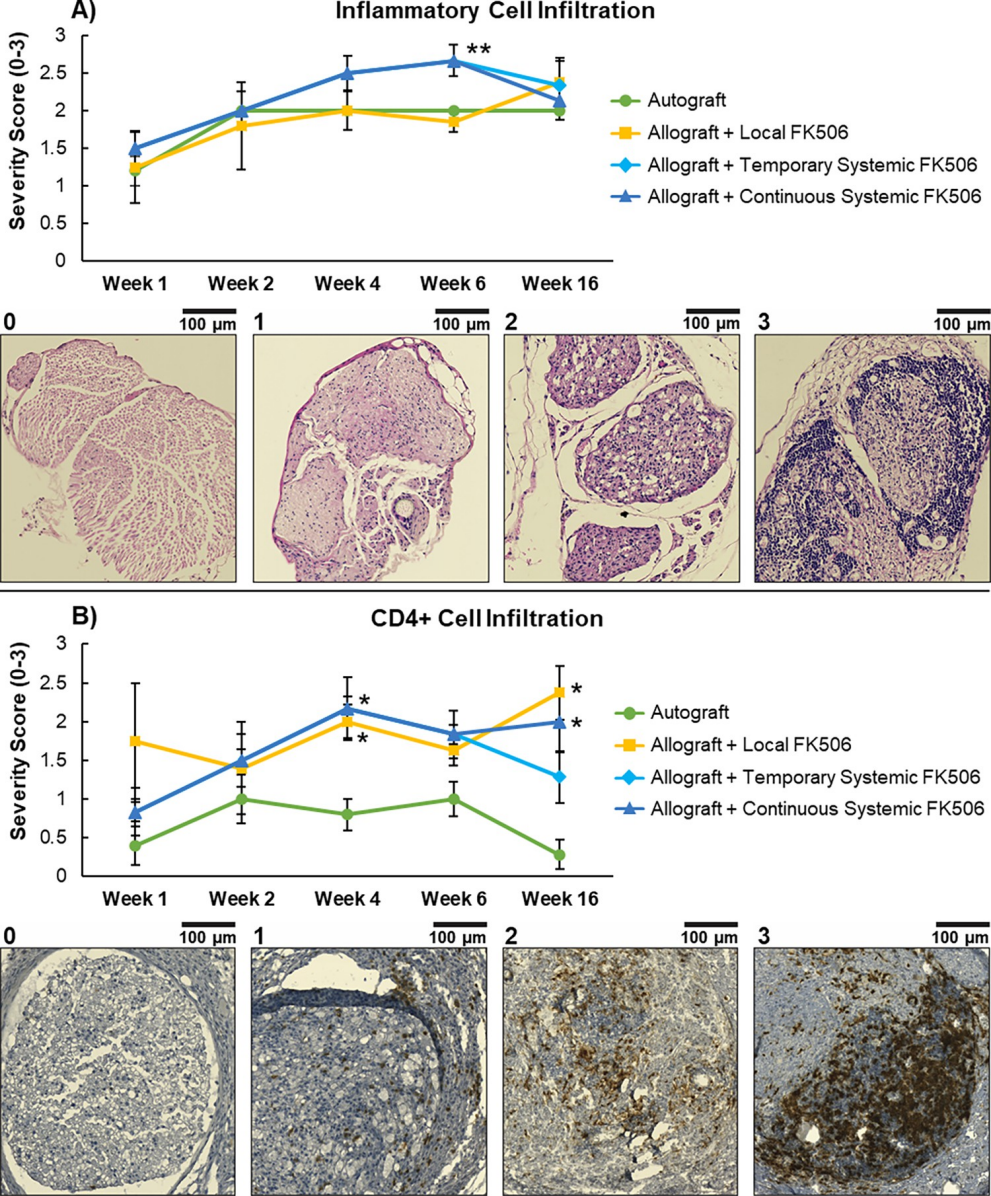
Serial assessment of the immune response to the allografts with varying FK506 treatments and autograft control. (A) Assessment of inflammatory cell infiltration average severity score. Representative images of each score (0–3) are found below the graph. (B) Assessment of CD4+ lymphocyte infiltration average severity score. Representative images of each score (0–3) are found below graph. (* P < 0.05 vs Autograft group; ** P < 0.05 vs Autograft & Local FK506 group).

The sciatic nerve grafts were harvested from the animals immediately upon sacrifice based upon the timeline in Fig 1. The nerve grafts were fixed in formalin for 24 h and then an approximately 5 mm portion from the center of the nerve graft was cut an embedded in paraffin. 5 μm sections were stained with hematoxylin and eosin (H&E) and imaged with light microscopy using an Axio Scan.Z1 (ZEISS, Oberkochen, BW, Germany). H&E stained paraffin sections were scored for inflammatory cell infiltration based on a 0–3 scale: 0 = normal, 1 = mild, 2 = moderate, 3 = severe (see Fig 1A for representative images of severity scale) [41].

### Immunohistochemical assessment of CD4+ cell infiltration

5 μm sections were taken from the paraffin embedded nerve graft tissue described above and immunohistochemically (IHC) stained for CD4+ lymphocytes. Slides were deparaffinized and an antigen retrieval step was performed with 6.0 pH sodium citrate for 20 minutes. The slides were then stained with a rabbit monoclonal anti-mouse CD4+ primary antibody ab183685 at a 1:500 dilution (Abcam, Cambridge, MA, USA) and then a polyclonal goat anti-rabbit HRP antibody (R&D Systems, Minneapolis, MN, USA) was used for secondary staining. The slides were counterstained with hematoxylin. The slides were imaged with light microscopy using an Axio Scan.Z1 (ZEISS, Oberkochen, BW, Germany) and were scored for CD4+ cell infiltration severity based on a 0–3 scale: 0 = normal, 1 = mild, 2 = moderate, 3 = severe [41].

### Nerve histomorphometry

A 5mm portion of the sciatic nerve immediately distal to the distal suture line of the nerve graft was harvested at the time of sacrifice. The nerve tissues were fixed in formalin for 24 h and then postfixed in 2% osmium tetroxide solution (Electron Microscopy Sciences, Hatfield, PA, USA) for 60 min, dehydrated, and embedded into Epon 812 resin (Electron Microscopy Sciences, Hatfield, PA, USA). 0.5 μm sections were stained with toluidine blue and imaged via light microscopy with a ZEISS AxioScan.Z1 (Oberkochen, Germany). Analysis was performed using ImageJ to determine nerve fascicle area, axon density, and total number of myelinated axons. Stereological techniques were used to obtain unbiased representations of the total number of myelinated axons and axon diameter per cross section [36, 42].

### Gastrocnemius muscle mass recovery assessment

The gastrocnemius muscle of both hind legs was harvested immediately after sacrifice by careful dissection at the tendinous origin and insertion points. The muscles were weighed and the relative muscle mass of the experimental leg was calculated by comparing the weight to the contralateral side: Relative % Gastrocnemius Muscle Mass = (Mass_Experimental_/Mass_Contralateral_) x 100.

### Statistical analysis

All results are presented as mean ± standard error. Significance level was set at p < 0.05. SPSS Statistics software (IBM, Armonk, NY, USA) was used for all statistical analysis. The inflammatory cell and CD4+ T-cell infiltrate severity score data was analyzed with a Kruskal-Wallis test with a pairwise comparison post-hoc test. The gastrocnemius muscle mass recovery, nerve histomorphometry, and ladder rung assay data was analyzed with a one-way analysis of variance with a Student’s t-test post-hoc test for group comparisons.

## Results

### Serial histological assessment of immune response

**Fig 2:**
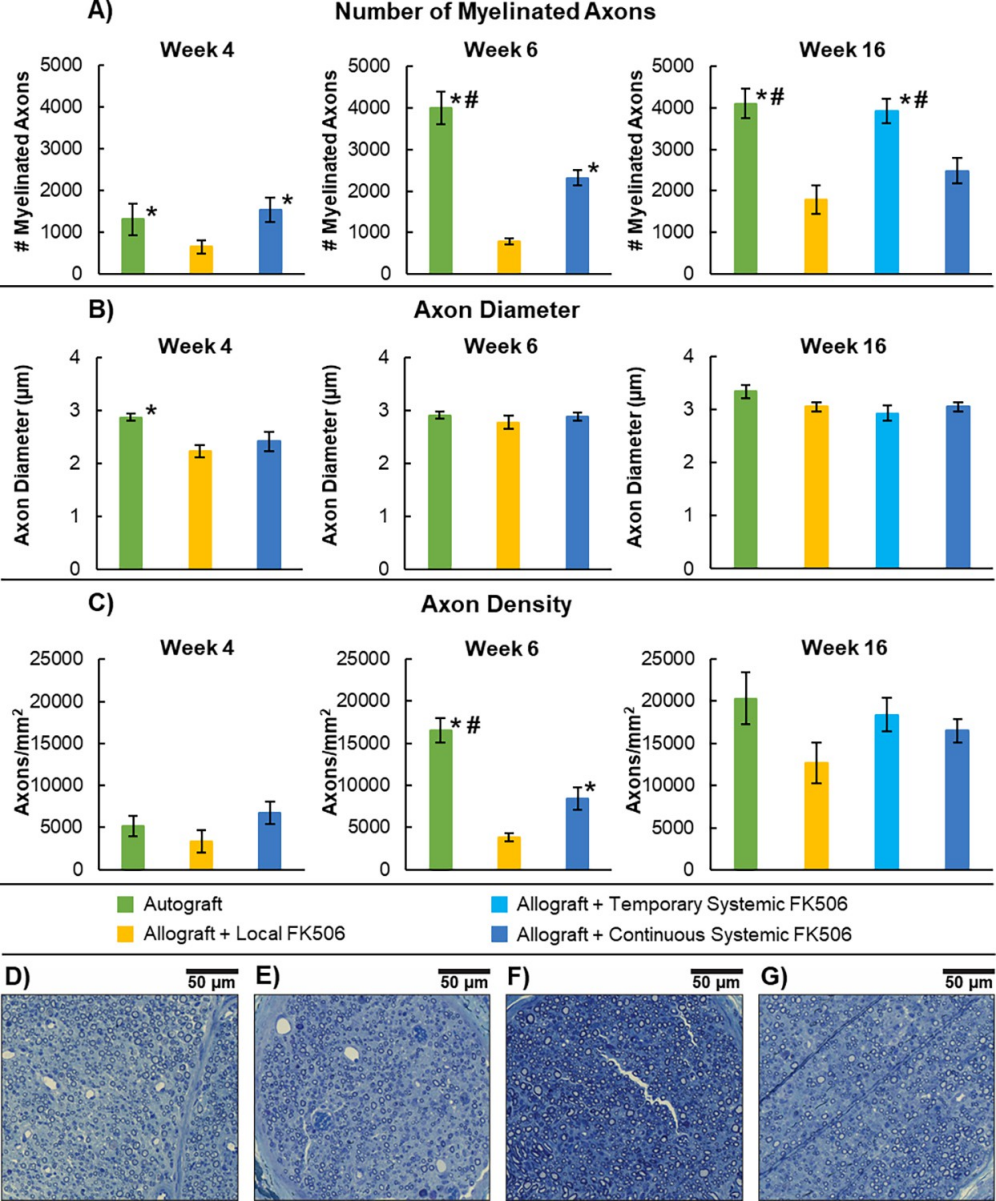
Nerve histomorphometry from tissue sections distal to the graft. (A) Average total number of myelinated axons. (B) Average myelinated axon diameter. (C) Average myelinated axon density. (D-G) Representative images from each group: (D) Autograft, (E) Allograft + Local FK506, (F) Allograft + Temporary FK506, and (G) Allograft + Continuous FK506. (* P < 0.05 vs Local FK506 group; # P < 0.05 Continuous FK506).

Serial histological assessment of inflammatory and CD4+ cell infiltration was performed to determine the immune response time course to allogeneic nerve grafts with either systemic or local delivery of FK506 immunosuppression. Since inflammatory cells and CD4+ lymphocytes have been implicated as critical role players in Wallerian degeneration and nerve regeneration, the autograft control group was used to determine the native inflammatory and CD4+ cell baselines in a nerve injury scenario [43, 44]. See Fig 1A for inflammatory cell infiltrate severity score data. At week 1, all groups had similar inflammatory cell infiltration severity scores in the mild-moderate. At week 2, all groups had similar inflammatory cell infiltration severity scores which increased to the moderate range. At week 4, the Continuous Systemic FK506 group began to diverge with an increase in severity, however, no statistically significant differences were found. At week 6, the heightened average inflammatory severity score of the Continuous Systemic FK506 group (2.67±0.21) was now significantly (p<0.05) greater than the Autograft (2.00±0.00) and Local FK506 (1.86±0.25) groups. At week 16, the average inflammatory cell infiltration severity score of the Continuous Systemic and Temporary Systemic FK506 groups decreased in intensity and converged with the Autograft and Local FK506 groups. The inflammatory cell infiltration data indicates that there is a moderate severity inflammatory cell response during natural Wallerian degeneration (see Autograft group Fig 2A) and that the systemically-delivered FK506 group (Continuous Systemic FK506) saw a significant increase in severity that at week 6. However, by week 16, all the groups converged and were not statistically different. This result shows that the allogeneic nerve graft with systemic treatment saw a temporary heightened inflammatory cell response that took around 6 weeks to develop, but returned to normal by week 16. See Fig 1B for CD4+ cell infiltrate severity score data. At week 1, there was a broad range of average CD4+ cell infiltration severity scores across the groups in the normal-moderate range, however, no statistically significant differences were found. At week 2, the range of average CD4+ cell infiltration severity scores across the groups tightened to the mild-moderate range with no statistically significant differences. At week 4, the Continuous Systemic FK506 (2.17±0.16) and Local FK506 (2.00±0.22) groups’ average CD4+ cell infiltration severity scores were significantly (p<0.05) greater than the Autograft group (0.80±0.200). At week 6, the Systemic FK506 and Local FK506 group’s average CD4+ cell infiltrate severity scores remained greater than the autograft, though no statistically significant differences were found. At week 16, the Continuous Systemic FK506 group (2.00±0.33) and Local FK506 group (2.38±0.35) had the most severe CD4+ cell infiltration scores and we’re significantly (p<0.05) greater than the Autograft group (0.286±0.18), and the Temporary Systemic FK506 group was found to be not statistically different than any of the groups. The CD4+ cell infiltration data indicates that the animals with allogeneic nerve grafts with either systemic or local FK506 immunosuppressive treatment, saw an increase in CD4+ cell infiltration. However, when systemic FK506 immunosuppression was discontinued at week 6, the Temporary Systemic FK506 group CD4+ cell infiltration score reduced and was not significantly different than the Autograft. Overall, the histological assessment of immune response data suggests that the groups with allogeneic nerve grafts induce a relatively greater immune response than the autograft group, but it took around week 4 to develop. Additionally, the data suggests that an acute period of allogeneic tissue rejection occurs when FK506 treatment is discontinued, but the rejection subsides by week 16.

### Nerve histomorphometry

Nerve histomorphometric analysis was performed to assess nerve regeneration across the various nerve grafts and treatments. Total number of myelinated axons, axon diameter, and axon density was quantified for each animal sacrificed at weeks 4, 6, and 16 (Fig 2). At week 4, the Autograft and Continuous Systemic FK506 group had significantly (p<0.05) greater average number of myelinated axons than the Local FK506 group; the Autograft, Local FK506, and Continuous Systemic FK506 groups had an average total number of myelinated axons of 1,310±380, 650±150, and 1,540±290, respectively. At week 6, the Autograft group had significantly (p<0.05) greater average number of myelinated axons than both the Local FK506 and Continuous Systemic FK506 groups, and the Continuous Systemic FK506 group also had significantly (p<0.05) greater number of myelinated axons than the Local FK506 group; the Autograft, Local FK506, and Continuous Systemic FK506 groups had an average total number of myelinated axons of 4,000 ± 400, 800±70, and 2,320±190, respectively. At week 16, the Autograft and Temporary Systemic FK506 groups had significantly (p<0.05) greater average number of myelinated axons than both the Local FK506 and Continuous Systemic FK506 groups; the Autograft, Local FK506, Temporary Systemic FK506, and Continuous Systemic FK506 groups had an average total number of myelinated axons of 4,100 ± 400, 1,800±280, 3,930±290, and 2,480±300, respectively. At week 4, the Autograft group (2.87±0.069μm) had significantly greater average axon diameter than the Local FK506 group (2.23±0.121μm). At week 6, no significant differences of average axon diameter were found. At week 16, no significant differences of average axon diameter were found. At week 4, no significant differences of average myelinated axon density were found. At week 6, the Autograft group (16,510±1,450 axons/mm^2^) had significantly (p<0.05) greater average myelinated axon density than both the Local FK506 (3,860±490 axons/mm^2^) and Continuous Systemic FK506 groups (8,400±1,320 axons/mm^2^), and the Continuous Systemic FK506 group also had significantly (p<0.05) greater myelinated axon density than the Local FK506 group. At week 16, no significant differences of average myelinated axon density were found, indicating that local delivery of FK506 had similar levels of immunosuppressive effects as the systemic delivery of FK506 in this study in regards to nerve histomorphometry. However, nerve histomorphometry outcomes were significantly lower than both the Autograft and Temporary Systemic FK506 group, suggesting that sustained FK506 treatment produces an inhibitory effect to nerve regeneration outcomes. Also, at week 16, the Autograft and Temporary Systemic FK506 group had no significant differences in nerve histomorphometry outcomes, suggesting that discontinuing FK506 immunosuppression after nerve regeneration has passed through the allogeneic nerve graft will produce nerve regeneration outcomes similar to the Autograft.

### Gastrocnemius muscle mass recovery

**Fig 3:**
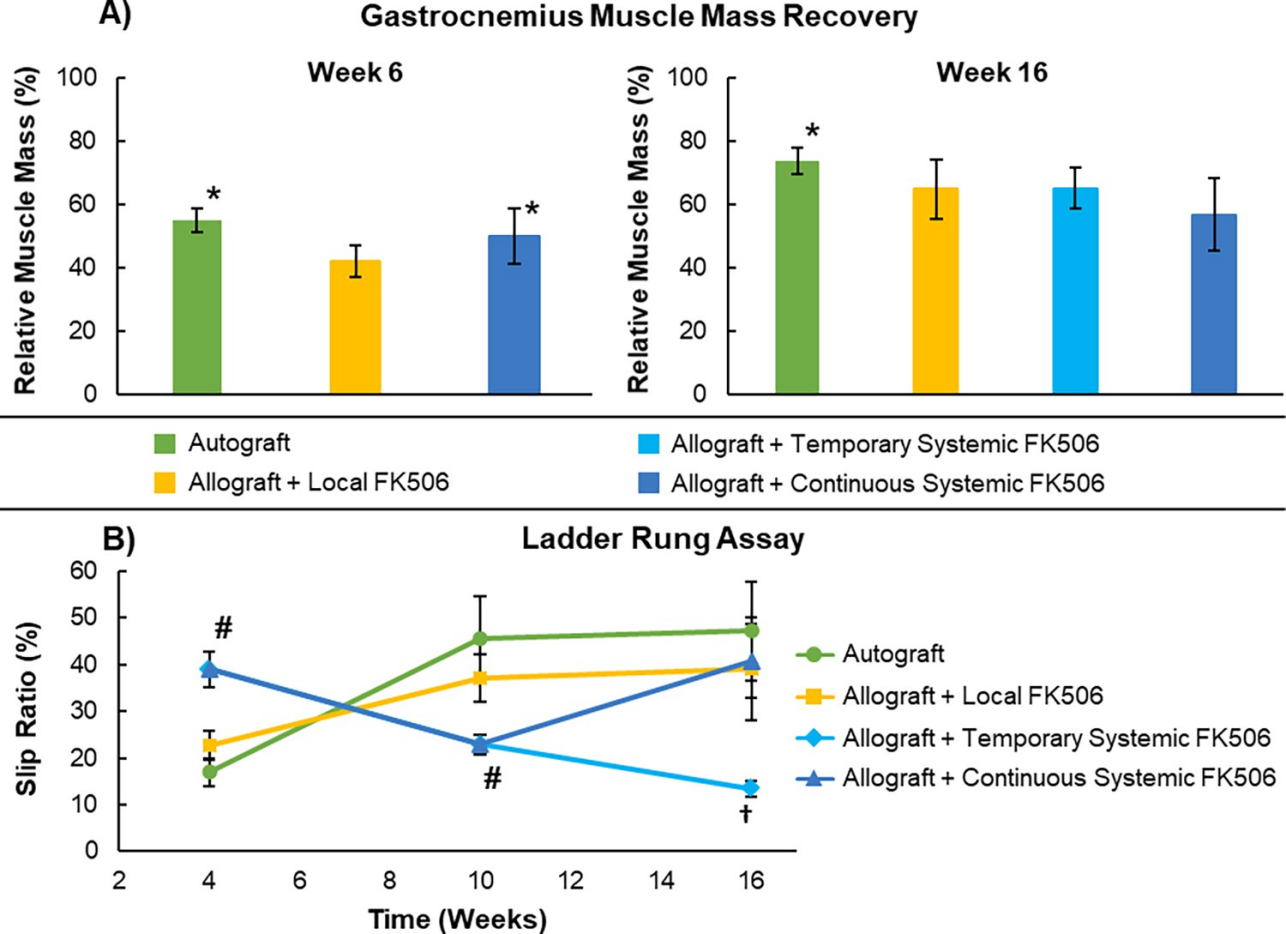
Serial assessment of functional recovery. (A) Average gastrocnemius muscle mass recovery at various timepoints (B) Average slip ratio during the ladder rung assay at various timepoints (* P < 0.05 vs Local FK506 group; # P < 0.05 Local FK506 & Autograft groups; † P<0.05 vs all other groups).

Muscle mass recovery after denervation is a gross measure of motor neuron end-target regeneration [45]. The relative gastrocnemius muscle mass was measured upon harvest at weeks 6 and 16 to compare nerve regeneration across groups, see Fig 3A. At week 6, the Autograft (55.1±1.45%) and Continuous Systemic FK506 (49.9±3.32%) groups had significantly (p<0.05) greater relative gastrocnemius muscle mass recovery than the Local FK506 (42.0±1.89%) group. At week 16, the Autograft group had significantly (p<0.05) greater relative gastrocnemius muscle mass recovery than all the other groups. The relative gastrocnemius muscle mass recovery data suggests that the autograft produces the best muscle mass recovery by week 16 and that Local FK506 delivery produces statistically similar muscle mass recovery as both continuously and temporarily systemically delivery FK506.

### Locomotion functional assessment: The ladder rung assay

The ladder rung assay was used to assess functional recovery after nerve injury. Skilled locomotion was assessed by measuring the slip ratio (missed steps / total steps) of the animals crossing a horizontal ladder. The ladder rung assay data can be found in Fig 3B. At week 4, the Continuous Systemic FK506 (39.0±3.86%) group had a significantly (p<0.05) greater slip ratio than both the Autograft (16.9±2.97%) and Local FK506 (22.7±5.11%) groups. At week 10, the Continuous Systemic FK506 (22.9±2.07%) group had a significantly (p<0.05) lower slip ratio than both the Autograft (45.7±8.96) and Local FK506 (37.1±2.67%) groups. At week 16, the Temporary FK506 (13.5±1.67%) group had a significantly (p<0.05) lower slip ratio than the Autograft, Continuous Systemic FK506, and Local FK506 groups. The Autograft and Local FK506 groups had better skilled locomotion than the Continuous FK506 group initially at week 4, but then progressively became worse for the rest of the study period. The Continuous FK506 group initially had the worst slip ratio at week 4, then improved by week 10, and then worsened again to its final point at week 16. At the end of the study, the Autograft, Continuous FK506, and Local FK506 groups had statistically similar skilled locomotion performance. This indicates that local delivery of FK506 provides similar functional recovery outcomes as systemically delivered FK506, which was statistically similar to the Autograft. Additionally, the Temporary Systemic FK506 group had significantly better skilled locomotion performance than all the other groups at the final week 16 timepoint, which indicates that discontinuing FK506 after nerve regeneration has passed through nerve graft might have an added benefit in sensorimotor recovery.

## Discussion

Local delivery of immunosuppressant drugs is a novel field of research with only a limited number of studies being published to date [28–32, 35]. If proven effective, localized immunosuppressive therapy could prevent allograft tissue rejection while avoiding the harsh side-effects associated with systemic immunosuppressive therapy. Currently, systemic immunosuppression is used with solid tissue allograft transplantation as a treatment strategy primarily for life-threatening diseases (e.g. cardiac, pulmonary, and renal failure). However, in less severe disease scenarios, such as peripheral nerve injury, the potentially lethal side-effects associated with systemic immunosuppression has prevented the use of nerve allografts as a treatment strategy. Numerous studies have shown that nerve allograft repair with systemic FK506 immunosuppression provides similar levels of nerve regeneration as the autograft [21, 22, 24, 46–48]. The hypothesis is that immunosuppression only needs to be maintained temporarily to allow nerve regeneration to pass through the graft, then immunosuppression can be discontinued [21, 22, 24]. After discontinuation of immunosuppression, an acute period of rejection occurs, but then nerve regeneration outcomes return to levels similar of the autograft. In this study, using a previously developed FK506-delivering nerve conduit, we set out to determine if local delivery of FK506 to an allogeneic nerve graft could prevent allograft rejection long enough to allow axon regeneration to pass through the graft [36, 37]. The results from the study indicate that local delivery of FK506 can produce similar levels of immunosuppression as systemically delivered of FK506, and that nerve regeneration outcomes reach similar levels between local and systemically delivered FK506.

We serially assessed the immune response to the allogeneic nerve grafts with varying immunosuppressive treatments to determine if local delivery of FK506 could produce similar levels of immunosuppression as systemically delivered FK506 (Fig 1). An autograft control was used to determine a baseline immune response, since both inflammatory cells and CD4+ cells are key role players in the Wallerian degeneration and nerve regeneration processes after nerve injury [43, 44, 49–54]. The Continuous Systemic FK506 group received systemic FK506 delivery throughout the entirety of the study, and the Local FK506 group provided a sustained period of FK506 delivery that was predicted to provide therapeutic dosages for approximately 8 weeks. We found that the autograft control group had a moderate inflammatory cell infiltration and a mild CD4+ cell infiltration that peaked at week 2 and this was sustained for the remainder of the study (Fig 1). Both the Continuous Systemic and Local FK506 groups saw a heightened CD4+ cell presence that reached statistically different levels than the Autograft group at week 4. At week 16, there were no significant differences in inflammatory cell infiltration between all groups, however, the Local FK506 and Continuous Systemic FK506 groups both had significantly greater levels of CD4+ cell infiltrate than the Autograft group. These results indicate that the Local FK506 group provided similar immunosuppression levels as the Continuous FK506 group, and that even with immunosuppressive therapy, a heightened CD4+ cell response may be observed. Another important observation was that the Local FK506 group saw an increase in both inflammatory cell and CD4+ cell infiltration occurs in the period between week 6 and week 16, although, only the CD4+ cell infiltration was statistically significant. This late onset of increased immune cell presence observed in the Local FK506 group is likely due to a decreasing local FK506 concentration over time, which may have caused acute rejection period to occur around week 16.

In regards to the nerve regeneration outcomes at the end of the study, the Local FK506 and Continuous Systemic FK506 group had no statistical differences in the nerve histomorphometry, gastrocnemius muscle mass recovery, and ladder rung assay outcomes. This result indicates that similar levels of nerve regeneration were achieved between the groups receiving either local or systemic delivery of FK506. However, the Local FK506 and Continuous Systemic FK506 groups had significantly lower total number of myelinated axons than the Autograft and Temporary Systemic FK506 groups, and they also had lower gastrocnemius muscle mass recovery than the Autograft group. For the ladder rung assay at week 16, the Local FK506, Continuous FK506, and Autograft groups had no significant differences between groups, therefore there was no difference in functional recovery from the autograft. The observed elevated presence of CD4+ cells in the Continuous FK506 and Local FK506 group may be to the presence of acute rejection, which led to lower histomorphometry and muscle mass recovery outcomes than the autograft. It is also common for solid-organ transplants (e.g. renal, hepatic, cardiac, etc.) in the clinic to experience sub-clinical and clinical acute rejection even with immunosuppressive therapy, which is histologically described by increased mononuclear immune cell infiltration [55]. It can be postulated that if given more time, after FK506 delivery from the FK506-delivering nerve conduits completely ceases, the acute rejection period would subside and nerve regeneration outcomes would further increase to levels similar as the Temporary FK506 group.

Another interesting takeaway from the study is that the Temporary Systemic FK506 group achieved similar levels of nerve regeneration and significantly better skilled locomotion functional recovery than the Autograft group (Fig 3B). At the final timepoint, the Autograft group and the Temporary Systemic FK506 group had the highest levels of total myelinated axons with no significant differences found between the two groups. This correlates with the CD4+ cell infiltrate data, indicating that after discontinuation of FK506 at week 6, the allogeneic nerve tissue in the Temporary FK506 group goes through a period of acute rejection, which is resolved by week 16. After the acute rejection period, nerve regeneration proceeds to levels similar as the Autograft group. This result is similar to previous studies and affirms the hypothesis that temporary FK506 administration to nerve allografts can produce nerve regeneration levels similar to autograft treatment [21, 22, 24]. For the skilled locomotion ladder rung functional assay, the Temporary Systemic FK506 group had the best functional recovery, with significantly lower slip ratios than all the other groups. After reexamination of the video recordings of the animals performing the ladder assay, it was clear that the Temporary Systemic FK506 animals had much greater control of the injured hindlimb, and the animals of the other groups appeared to have a nociceptive hypersensitivity to the physical stimulus of touching the ladder rungs with the hindpaw. This nociceptive hypersensitivity was just qualitatively observed, not quantitatively assessed. It has been shown by others after peripheral nerve injury that rodents experience mechanical and thermal nociceptive hypersensitivity, and that superior nerve regeneration will have reduced nociceptive hypersensitivity [56]. The observed reduced hypersensitivity in the Temporary FK506 group compared to the Autograft group may be due to the added neurotrophic effects of FK506 [19, 57–61]. Temporary FK506 may have accelerated axon regeneration and increased sensory axon sprouting, leading to better overall sensorimotor functional recovery at the end of the study. Additionally, intraneural lymphocyte and macrophage induced inflammation has been implicated in causing neuropathic pain after peripheral nerve injury [51, 62]. It is possible that immunomodulation via FK506 or the acute period of intense immune response after FK506 discontinuation, produced a local cellular milieu that resulted in enhanced overall sensory functional outcomes. One recent study has reported that delivering lymphocyte cell therapy acutely after peripheral nerve injury improves nerve regeneration outcomes [63]. Since the immunosuppressive effects of FK506 are necessary to determine if localized immunosuppression can prevent allograft rejection, and FK506 has neurotrophic effects, it complicates the ability to differentiate which drug mechasnism effect is causing the enhanced nerve regeneration. Since functional outcomes in the Continous, Local FK506 group, and autograft group were similar, it is possible the neurotrophic effects of FK506 was the main driver nerve regeneration, not the immunosuppression of the allograft. Nerve regeneration outcomes are relatively satisfactory in mouse sciatic nerve gap models due to the relatively short distance nerve regeneration needs to traverse. The current study was not designed to elucidate between these potential mechanisms, and further investigation in a larger animal model is needed to determine the role of FK506 may play in sensorimotor functional recovery after allogeneic nerve grafting.

In recent years, there have been several studies investigating the efficacy of locally delivered FK506 immunosuppressive therapy to allogeneic tissue to prevent tissue rejection; however, they have primarily focused on delivering local immunosuppressive therapy to vascularized composite allografts [28–34]. To date, only one published study has attempted to assess the efficacy of locally delivered FK506 to prevent host rejection of a nerve allograft [28, 35]. All of these studies utilized an implantable polymeric-based drug delivery system that could provide sustained release of FK506 for weeks to months. These drug delivery systems are implanted into or adjacent to the allograft transplant and provide sustained immunosuppressant drug release directly to the allogeneic tissue. Unadkat et al. developed polyester-based FK506-loaded disks and implanted them into transplanted rat hindlegs, the animals receiving these disks had 100% allograft survival for more than 180 days [32]. Gajanayake et al. utilized a FK506-laden injectable hydrogel that prolonged rat hindlimb survival to greater than 100 days [34]. These polymeric-based implantable drug delivery systems show promise for prolonged survival of allografts via local delivery of immunosuppressive therapy, but they have not evaluated their system in nerve allograft repair scenarios. Zuo et al. applied their FK506-loaded polymeric microspheres entrapped into a fibrin hydrogel system adjacent to sciatic nerve allografts in a rat model. They found that allografts treated with their local FK506 delivery system had no significant differences in nerve histomorphometry compared to the autograft control group, and that the allografts treated with their local FK506 delivery system had significantly lower serum IL-12 cytokine concentrations than allografts without drug treatment. Our FK506-delivering nerve conduit has been shown to release FK506 for longer than 60 days, which may be useful in larger gap scenarios [36, 37]. However, the drug release kinetics may need to be tuned specifically for larger gaps in order to maintain an immunosuppressive therapeutic concentration of FK506 while the axonal regeneration passes through the entire allograft. Polyester-based depot drug delivery systems are highly tunable for drug release kinetics and further studies would be needed to determine optimal drug release kinetics for various nerve allograft repair scenarios [64].

## Conclusion

In conclusion, results from this study suggest that local delivery of FK506 to nerve allografts can provide similar immunosuppression and nerve regeneration outcomes as systemic FK506 delivery, and that temporary FK506 administration to a nerve allograft can produce similar nerve regeneration end-results as an autograft repair. Additionally, temporary FK506 delivery may provide an added benefit of enhanced sensorimotor functional recovery, which may be attributed to the added neurotrophic effects of FK506 early on in the regeneration process. Further studies are needed to determine the optimal local FK506 concentrations and duration of release for local immunosuppression in nerve allograft repair strategies.

## References

[1] Ruijs AC, Jaquet JB, Kalmijn S, Giele H, Hovius SE. Median and ulnar nerve injuries: a meta-analysis of predictors of motor and sensory recovery after modern microsurgical nerve repair. Plast Reconstr Surg. 2005;116(2):484–94; discussion 95–6. doi: 10.1097/01.prs.0000172896.86594.07 . 16079678

[2] Lee SK, Wolfe SW. Peripheral nerve injury and repair. J Am Acad Orthop Surg. 2000;8(4):243–52. doi: 10.5435/00124635-200007000-00005 . 10951113

[3] Terenghi G. Peripheral nerve injury and regeneration. Histol Histopathol. 1995;10(3):709–18. Epub 1995/07/01. . 7579822

[4] Ronald Deumens AB, Marcel F. Meek, Marco A.E. Marcus, Elbert A.J.Joosten, Joachim Weis, Gary A. Brook. Repairing injured peripheral nerves: Bridging the gap. Progress in Neurobiology. 2010;92(3):245–76. doi: 10.1016/j.pneurobio.2010.10.002 20950667

[5] Kehoe S, Zhang XF, Boyd D. FDA approved guidance conduits and wraps for peripheral nerve injury: A review of materials and efficacy. Injury. 2012;43(5):553–72. doi: 10.1016/j.injury.2010.12.030 WOS:000302267100005. 21269624

[6] Daly W, Yao L, Zeugolis D, Windebank A, Pandit A. A biomaterials approach to peripheral nerve regeneration: bridging the peripheral nerve gap and enhancing functional recovery. J R Soc Interface. 2012;9(67):202–21. Epub 2011/11/18. doi: 10.1098/rsif.2011.0438 ; PubMed Central PMCID: PMC3243399. 22090283 PMC3243399

[7] Grinsell D, Keating CP. Peripheral nerve reconstruction after injury: a review of clinical and experimental therapies. Biomed Res Int. 2014;2014:698256. doi: 10.1155/2014/698256 ; PubMed Central PMCID: PMC4167952. 25276813 PMC4167952

[8] M FG, M M, S H, Khan WS. Peripheral nerve injury: principles for repair and regeneration. Open Orthop J. 2014;8:199–203. Epub 2014/07/30. doi: 10.2174/1874325001408010199 ; PubMed Central PMCID: PMC4110386. 25067975 PMC4110386

[9] Schultz JD, Dodson TB, Meyer RA. Donor site morbidity of greater auricular nerve graft harvesting. J Oral Maxillofac Surg. 1992;50(8):803–5. Epub 1992/08/01. doi: 10.1016/0278-2391(92)90269-6 . 1634971

[10] Safa B, Buncke G. Autograft Substitutes: Conduits and Processed Nerve Allografts. Hand Clin. 2016;32(2):127–40. Epub 2016/04/21. doi: 10.1016/j.hcl.2015.12.012 . 27094886

[11] Lewin-Kowalik J, Marcol W, Kotulska K, Mandera M, Klimczak A. Prevention and management of painful neuroma. Neurol Med Chir (Tokyo). 2006;46(2):62–7; discussion 7–8. Epub 2006/02/25. doi: 10.2176/nmc.46.62 . 16498214

[12] Dagum AB. Peripheral nerve regeneration, repair, and grafting. J Hand Ther. 1998;11(2):111–7. Epub 1998/05/29. doi: 10.1016/s0894-1130(98)80007-0 . 9602967

[13] de Ruiter GC, Malessy MJ, Yaszemski MJ, Windebank AJ, Spinner RJ. Designing ideal conduits for peripheral nerve repair. Neurosurg Focus. 2009;26(2):E5. Epub 2009/05/14. doi: 10.3171/FOC.2009.26.2.E5 ; PubMed Central PMCID: PMC2978041. 19435445 PMC2978041

[14] Pinho AC, Fonseca AC, Serra AC, Santos JD, Coelho JF. Peripheral Nerve Regeneration: Current Status and New Strategies Using Polymeric Materials. Adv Healthc Mater. 2016;5(21):2732–44. Epub 2016/09/08. doi: 10.1002/adhm.201600236 . 27600578

[15] Giusti G, Willems WF, Kremer T, Friedrich PF, Bishop AT, Shin AY. Return of motor function after segmental nerve loss in a rat model: comparison of autogenous nerve graft, collagen conduit, and processed allograft (AxoGen). J Bone Joint Surg Am. 2012;94(5):410–7. Epub 2012/03/09. doi: 10.2106/JBJS.K.00253 . 22398734

[16] Moore AM, MacEwan M, Santosa KB, Chenard KE, Ray WZ, Hunter DA, et al. Acellular nerve allografts in peripheral nerve regeneration: a comparative study. Muscle Nerve. 2011;44(2):221–34. Epub 2011/06/11. doi: 10.1002/mus.22033 ; PubMed Central PMCID: PMC3136642. 21660979 PMC3136642

[17] Evans PJ, Midha R, Mackinnon SE. The peripheral nerve allograft: a comprehensive review of regeneration and neuroimmunology. Prog Neurobiol. 1994;43(3):187–233. Epub 1994/06/01. doi: 10.1016/0301-0082(94)90001-9 . 7816927

[18] Lin MY, Manzano G, Gupta R. Nerve allografts and conduits in peripheral nerve repair. Hand Clin. 2013;29(3):331–48. Epub 2013/07/31. doi: 10.1016/j.hcl.2013.04.003 . 23895714

[19] Konofaos P, Terzis JK. FK506 and nerve regeneration: past, present, and future. J Reconstr Microsurg. 2013;29(3):141–8. Epub 2013/01/17. doi: 10.1055/s-0032-1333314 . 23322540

[20] Chapman JR. Chronic calcineurin inhibitor nephrotoxicity-lest we forget. Am J Transplant. 2011;11(4):693–7. Epub 2011/03/31. doi: 10.1111/j.1600-6143.2011.03504.x . 21446974

[21] Mackinnon SE, Doolabh VB, Novak CB, Trulock EP. Clinical outcome following nerve allograft transplantation. Plast Reconstr Surg. 2001;107(6):1419–29. Epub 2001/05/04. doi: 10.1097/00006534-200105000-00016 . 11335811

[22] Udina E, Gold BG, Navarro X. Comparison of continuous and discontinuous FK506 administration on autograft or allograft repair of sciatic nerve resection. Muscle Nerve. 2004;29(6):812–22. doi: 10.1002/mus.20029 . 15170614

[23] Jensen JN, Brenner MJ, Tung TH, Hunter DA, Mackinnon SE. Effect of FK506 on peripheral nerve regeneration through long grafts in inbred swine. Ann Plast Surg. 2005;54(4):420–7. Epub 2005/03/24. doi: 10.1097/01.sap.0000151461.60911.c0 . 15785285

[24] Auba C, Hontanilla B, Arcocha J, Gorria O. Peripheral nerve regeneration through allografts compared with autografts in FK506-treated monkeys. J Neurosurg. 2006;105(4):602–9. Epub 2006/10/19. doi: 10.3171/jns.2006.105.4.602 . 17044565

[25] Midha R, Mackinnon SE, Evans PJ, Best TJ, Hare GM, Hunter DA, et al. Comparison of regeneration across nerve allografts with temporary or continuous cyclosporin A immunosuppression. J Neurosurg. 1993;78(1):90–100. Epub 1993/01/01. doi: 10.3171/jns.1993.78.1.0090 . 8416248

[26] Myckatyn TM, Hunter DA, Mackinnon SE. The effects of cold preservation and subimmunosuppressive doses of FK506 on axonal regeneration in murine peripheral nerve isografts. Can J Plast Surg. 2003;11(1):15–22. Epub 2003/04/01. doi: 10.1177/229255030301100110 ; PubMed Central PMCID: PMC3792774. 24115844 PMC3792774

[27] Buttemeyer R, Rao U, Jones NF. Peripheral nerve allograft transplantation with FK506: functional, histological, and immunological results before and after discontinuation of immunosuppression. Ann Plast Surg. 1995;35(4):396–401. Epub 1995/10/01. . 8585683

[28] Zuo KJM, MASc; Shafa Golsa BSc; Chan Katelyn B.Eng BioSci; Zhang Jennifer MD, PhD; Tajdaran Kasra MASc, PhD; Gordon Tessa PhD; Borschel Gregory MD, FACS. Abstract 10: Local FK506 Drug Delivery Enhances Nerve Regeneration Through Fresh Nerve Allografts. Plastic and Reconstructive Surgery—Global Open. 2020;8(4S):7–8. doi: 10.1097/01.GOX.0000667104.40941.7e

[29] Gama AR, Ng ZY, Shanmugarajah K, Mastroianni M, Randolph MA, Lellouch AG, et al. Local Immunosuppression for Vascularized Composite Allografts: Application of Topical FK506-TyroSpheres in a Non-Human Primate Model. J Burn Care Res. 2020. Epub 2020/05/01. doi: 10.1093/jbcr/iraa062 . 32352521

[30] Taddeo A, Tsai C, Vogelin E, Rieben R. Novel targeted drug delivery systems to minimize systemic immunosuppression in vascularized composite allotransplantation. Curr Opin Organ Transplant. 2018;23(5):568–76. Epub 2018/08/04. doi: 10.1097/MOT.0000000000000564 . 30074507

[31] Dzhonova DV, Olariu R, Leckenby J, Banz Y, Prost JC, Dhayani A, et al. Local Injections of Tacrolimus-loaded Hydrogel Reduce Systemic Immunosuppression-related Toxicity in Vascularized Composite Allotransplantation. Transplantation. 2018;102(10):1684–94. Epub 2018/05/26. doi: 10.1097/TP.0000000000002283 . 29794937

[32] Unadkat JV, Schnider JT, Feturi FG, Tsuji W, Bliley JM, Venkataramanan R, et al. Single Implantable FK506 Disk Prevents Rejection in Vascularized Composite Allotransplantation. Plast Reconstr Surg. 2017;139(2):403e–14e. Epub 2017/01/26. doi: 10.1097/PRS.0000000000002951 . 28121868

[33] Wang S, Xiong Y, Wang Y, Chen J, Yang J, Sun B. Evaluation of PLGA microspheres with triple regimen on long-term survival of vascularized composite allograft—an experimental study. Transpl Int. 2020;33(4):450–61. Epub 2020/01/14. doi: 10.1111/tri.13574 . 31930539

[34] Gajanayake T, Olariu R, Leclere FM, Dhayani A, Yang Z, Bongoni AK, et al. A single localized dose of enzyme-responsive hydrogel improves long-term survival of a vascularized composite allograft. Sci Transl Med. 2014;6(249):249ra110. Epub 2014/08/15. doi: 10.1126/scitranslmed.3008778 . 25122638

[35] Zuo KJ, Shafa G, Chan K, Zhang J, Hawkins C, Tajdaran K, et al. Local FK506 drug delivery enhances nerve regeneration through fresh, unprocessed peripheral nerve allografts. Exp Neurol. 2021;341:113680. Epub 2021/03/07. doi: 10.1016/j.expneurol.2021.113680 . 33675777

[36] Davis B, Hilgart D, Erickson S, Labroo P, Burton J, Sant H, et al. Local FK506 delivery at the direct nerve repair site improves nerve regeneration. Muscle Nerve. 2019;60(5):613–20. Epub 2019/08/10. doi: 10.1002/mus.26656 . 31397908

[37] Davis B, Wojtalewicz S, Labroo P, Shea J, Sant H, Gale B, et al. Controlled release of FK506 from micropatterned PLGA films: potential for application in peripheral nerve repair. Neural Regen Res. 2018;13(7):1247–52. Epub 2018/07/22. doi: 10.4103/1673-5374.235063 ; PubMed Central PMCID: PMC6065245. 30028334 PMC6065245

[38] Katari SR, Magnone M, Shapiro R, Jordan M, Scantlebury V, Vivas C, et al. Clinical features of acute reversible tacrolimus (FK 506) nephrotoxicity in kidney transplant recipients. Clin Transplant. 1997;11(3):237–42. Epub 1997/06/01. ; PubMed Central PMCID: PMC2967284. 9193849 PMC2967284

[39] McDiarmid SV, Busuttil RW, Ascher NL, Burdick J, D’Alessandro AM, Esquivel C, et al. FK506 (tacrolimus) compared with cyclosporine for primary immunosuppression after pediatric liver transplantation. Results from the U.S. Multicenter Trial. Transplantation. 1995;59(4):530–6. Epub 1995/02/27. . 7533345

[40] Kemp SW, Alant J, Walsh SK, Webb AA, Midha R. Behavioural and anatomical analysis of selective tibial nerve branch transfer to the deep peroneal nerve in the rat. Eur J Neurosci. 2010;31(6):1074–90. Epub 2010/04/10. doi: 10.1111/j.1460-9568.2010.07130.x . 20377620

[41] Davis B, Erickson S, Wojtalewicz S, Simpson A, Metcalf C, Sant H, et al. Entrapping bupivacaine-loaded emulsions in a crosslinked-hydrogel increases anesthetic effect and duration in a rat sciatic nerve block model. Int J Pharm. 2020;588:119703. Epub 2020/08/03. doi: 10.1016/j.ijpharm.2020.119703 . 32739385

[42] Mayhew TM. An Efficient Sampling Scheme for Estimating Fiber Number from Nerve Cross-Sections—the Fractionator. J Anat. 1988;157:127–34. WOS:A1988N249800013. 3198473 PMC1261946

[43] Chen P, Piao X, Bonaldo P. Role of macrophages in Wallerian degeneration and axonal regeneration after peripheral nerve injury. Acta Neuropathol. 2015;130(5):605–18. Epub 2015/10/01. doi: 10.1007/s00401-015-1482-4 . 26419777

[44] Dubovy P, Jancalek R, Kubek T. Role of inflammation and cytokines in peripheral nerve regeneration. Int Rev Neurobiol. 2013;108:173–206. Epub 2013/10/03. doi: 10.1016/B978-0-12-410499-0.00007-1 . 24083435

[45] Navarro X. Functional evaluation of peripheral nerve regeneration and target reinnervation in animal models: a critical overview. Eur J Neurosci. 2016;43(3):271–86. doi: 10.1111/ejn.13033 . 26228942

[46] Ishida O, Daves J, Tsai TM, Breidenbach WC, Firrell J. Regeneration following rejection of peripheral nerve allografts of rats on withdrawal of cyclosporine. Plast Reconstr Surg. 1993;92(5):916–26. Epub 1993/10/01. . 8415974

[47] Mackinnon SE, Midha R, Bain J, Hunter D, Wade J. An assessment of regeneration across peripheral nerve allografts in rats receiving short courses of cyclosporin A immunosuppression. Neuroscience. 1992;46(3):585–93. Epub 1992/01/01. doi: 10.1016/0306-4522(92)90146-s . 1545911

[48] Mackinnon SE, Hudson AR, Bain JR, Falk RE, Hunter DA. The peripheral nerve allograft: an assessment of regeneration in the immunosuppressed host. Plast Reconstr Surg. 1987;79(3):436–46. Epub 1987/03/01. . 3823218

[49] Gaudet AD, Popovich PG, Ramer MS. Wallerian degeneration: Gaining perspective on inflammatory events after peripheral nerve injury. J Neuroinflamm. 2011;8. doi: 10.1186/1742-2094-8-110 WOS:000295513800002. 21878126 PMC3180276

[50] Bombeiro AL, Santini JC, Thome R, Ferreira ERL, Nunes SLO, Moreira BM, et al. Enhanced Immune Response in Immunodeficient Mice Improves Peripheral Nerve Regeneration Following Axotomy. Front Cell Neurosci. 2016;10. ARTN 151. doi: 10.3389/fncel.2016.00151 WOS:000377666600001. 27378849 PMC4905955

[51] Moalem G, Xu K, Yu L. T lymphocytes play a role in neuropathic pain following peripheral nerve injury in rats. Neuroscience. 2004;129(3):767–77. doi: 10.1016/j.neuroscience.2004.08.035 WOS:000225511000026. 15541898

[52] Deng Pan DAH, Lauren Schellhardt, Sally Jo, Katherine B. Santosa, Ellen L. Larson AGF, Alison K. Snyder-Warwick, Susan E. Mackinnon, Wood MD. The accumulation of T cells within acellular nerve allografts is length-dependent and critical for nerve regeneration. Experimental Neurology. 2019;318:216–31. doi: 10.1016/j.expneurol.2019.05.009 31085199 PMC6605105

[53] Cattin AL, Burden JJ, Van Emmenis L, Mackenzie FE, Hoving JJ, Garcia Calavia N, et al. Macrophage-Induced Blood Vessels Guide Schwann Cell-Mediated Regeneration of Peripheral Nerves. Cell. 2015;162(5):1127–39. Epub 2015/08/19. doi: 10.1016/j.cell.2015.07.021 ; PubMed Central PMCID: PMC4553238. 26279190 PMC4553238

[54] Epelman S, Liu PP, Mann DL. Role of innate and adaptive immune mechanisms in cardiac injury and repair. Nat Rev Immunol. 2015;15(2):117–29. Epub 2015/01/24. doi: 10.1038/nri3800 ; PubMed Central PMCID: PMC4669103. 25614321 PMC4669103

[55] Bergler T, Jung B, Bourier F, Kuhne L, Banas MC, Rummele P, et al. Infiltration of Macrophages Correlates with Severity of Allograft Rejection and Outcome in Human Kidney Transplantation. PLoS One. 2016;11(6):e0156900. Epub 2016/06/11. doi: 10.1371/journal.pone.0156900 ; PubMed Central PMCID: PMC4902310. 27285579 PMC4902310

[56] Annika B. Malmberg AIB. Partial sciatic nerve injury in the mouse as a model of neuropathic pain: behavioral and neuroanatomical correlates. Pain. 1998;76(1–2):215–22. doi: 10.1016/S0304-3959(98)00045-1 9696476

[57] Udina E, Ceballos D, Gold BG, Navarro X. FK506 enhances reinnervation by regeneration and by collateral sprouting of peripheral nerve fibers. Exp Neurol. 2003;183(1):220–31. doi: 10.1016/s0014-4886(03)00173-0 . 12957505

[58] Jost SC, Doolabh VB, Mackinnon SE, Lee M, Hunter D. Acceleration of peripheral nerve regeneration following FK506 administration. Restor Neurol Neurosci. 2000;17(1):39–44. Epub 2001/08/08. . 11490076

[59] Doolabh VB, Mackinnon SE. FK506 accelerates functional recovery following nerve grafting in a rat model. Plast Reconstr Surg. 1999;103(7):1928–36. doi: 10.1097/00006534-199906000-00018 . 10359255

[60] Lee M, Doolabh VB, Mackinnon SE, Jost S. FK506 promotes functional recovery in crushed rat sciatic nerve. Muscle Nerve. 2000;23(4):633–40. doi: 10.1002/(sici)1097-4598(200004)23:4&lt;633::aid-mus24&gt;3.0.co;2-q . 10716776

[61] Gold BG, Storm-Dickerson T, Austin DR. The immunosuppressant FK506 increases functional recovery and nerve regeneration following peripheral nerve injury. Restor Neurol Neurosci. 1994;6(4):287–96. Epub 1994/01/01. doi: 10.3233/RNN-1994-6404 . 21551759

[62] Kobayashi Y, Kiguchi N, Maeda T, Kishioka S. The crosstalk between macrophages and T-lymphocytes plays a crucial role in neuropathic pain. J Pharmacol Sci. 2012;118:65p-p. WOS:000301883300195.

[63] Bombeiro AL, Lima BHM, Bonfanti AP, Oliveira ALR. Improved mouse sciatic nerve regeneration following lymphocyte cell therapy. Mol Immunol. 2020;121:81–91. Epub 2020/03/17. doi: 10.1016/j.molimm.2020.03.003 . 32172028

[64] Tyler B, Gullotti D, Mangraviti A, Utsuki T, Brem H. Polylactic acid (PLA) controlled delivery carriers for biomedical applications. Adv Drug Deliv Rev. 2016;107:163–75. Epub 20160715. doi: 10.1016/j.addr.2016.06.018 . 27426411

